# Concordance between Genotypic and Phenotypic Drug-Resistant Profiles of *Shigella* Isolates from Taiyuan City, Shanxi Province, China, 2005 to 2016

**DOI:** 10.1128/spectrum.00119-23

**Published:** 2023-05-30

**Authors:** Mimi Kong, Chunmei Liu, Yang Xu, Jitao Wang, Dong Jin

**Affiliations:** a Department of Epidemiology, School of Public Health, Shanxi Medical University, Taiyuan City, Shanxi Province, China; b Department of Microbiology Test, Taiyuan Center for Disease Control and Prevention, Taiyuan City, Shanxi Province, China; c State Key Laboratory of Infectious Disease Prevention and Control, National Institute for Communicable Disease Control and Prevention, Chinese Center for Disease Control and Prevention, Changping, Beijing, China; Instituto de Higiene

**Keywords:** *Shigella* spp., antimicrobial resistance, phenotypic drug-resistant profile, genotypic drug-resistant profile, concordance analysis

## Abstract

Antimicrobial resistance in *Shigella* spp. is a global public health concern. In this study, the AMR phenotypic profiles of 10 kinds of antibiotics were compared with the genotypic profiles using genomic analysis of 218 *Shigella* isolates from Taiyuan City, Shanxi Province, China, 2005 to 2016. Core genome Multilocus Sequence Typing (cgMLST) based on the EnteroBase Escherichia/*Shigella* scheme was used to obtain the genetic relatedness of *Shigella* isolates. Multiple-drug resistance was observed in 96.79% *Shigella* spp., and the resistance to antimicrobial agents varied between S. flexneri and S. sonnei. The genotypic results correlated well with the phenotypic profiles with concordance rates of 96.42% and 94.50% in S. flexneri and S. sonnei isolates, respectively, from Taiyuan City, Shanxi Province. The sensitivity and specificity of the genotypic antimicrobial susceptibility testing (AST) were 97.56% and 95.34% for S. flexneri, and 95.65% and 93.31% for S. sonnei isolates, respectively. A discrepancy of genotypic and phenotypic AST results existed in some cephalosporin- and azithromycin-resistant *Shigella* isolates; there were no clear resistance patterns to predict ciprofloxacin resistance. There were major discrepancies between genotypic and phenotypic AST in the genotypically resistant but phenotypically susceptible isolates. The drug-resistance patterns and essential drug-resistance genes to predict the phenotypic drug-resistant profiles were the discrepancies between S. flexneri and S. sonnei isolates. Phylogenetic analysis showed that isolates of the same cluster but with different antibiotic-resistance gene patterns occurred because of the loss or gain of antibiotic-resistance genes located in the plasmids and multidrug-resistance islands.

**IMPORTANCE** Antimicrobial resistance in *Shigella* spp. has become a global public health concern. In this study, we identified the antimicrobial susceptibility testing (AST) characteristics based on genomic sequences of 218 *Shigella* isolates and analyzed the correlation between genotypic and phenotypic antibiotic resistance profiles of *Shigella* spp., especially for fluoroquinolone, macrolides, and third-generation cephalosporins. Our results show that the genotypic results correlated with the phenotypic profiles with concordance rates of 96.42% and 94.50% in S. flexneri and S. sonnei isolates, respectively. The drug-resistance patterns and essential drug-resistance genes to predict the phenotypic drug-resistant profiles of S. flexneri and S. sonnei isolates in Taiyuan city were distinct. The discrepancy between genotypic and phenotypic AST was considerable in the genotypically resistant but phenotypically susceptible isolates. The information on drug resistance and resistance genes in this study can offer more details on the prevalence of drug resistance of *Shigella* spp.

## INTRODUCTION

*Shigella* spp. are the causative agent of shigellosis in humans. Globally, there are an estimated 188 million cases of shigellosis, and most cases are immunodeficient patients and children <5 years (1). *Shigella* spp. are divided into four species/serogroups according to their surface antigenic and biochemical characteristics. The current global epidemiological burden for shigellosis can be mainly attributed to two species—S. flexneri and S. sonnei—which were conventionally associated with developing and developed regions, respectively (1, 2). Shigellosis is an important public health problem in China, and the prevalent species are different in different regions (3–5). In Shanxi province, the most prevalent *Shigella* species is S. sonnei, followed by S. flexneri (6). A study based on 11 years of active surveillance data showed that the proportion of *Shigella*-infections cases in diarrhea patients in Taiyuan city of Shanxi province was 8.4% during 2006–2015, and S. flexneri was the dominant species; however, S. sonnei showed a significant upward trend and gradually replaced S. flexneri as the dominant population (5).

Antibiotics are used to treat *Shigella* dysentery to hasten recovery and reduce the symptoms and risk of pathogen shedding (7). Per the WHO recommendation, ciprofloxacin was used as the first choice for treatment of dysentery in adults and children, and azithromycin and third-generation cephalosporins were considered second choices (8). With the widespread use of antibiotics, resistance is also a widespread phenomenon. Antibiotic-resistant *Shigella* spp., especially fluoroquinolone- and third-generation cephalosporins–resistant *Shigella*, have become a global public health concern (1). The emergence of antimicrobial resistance and multiple drug resistance (MDR) *Shigella* spp. limits future treatment options and enhances the prevalence of some clonal *Shigella* spp. (9, 10).

Conventional laboratory antimicrobial susceptibility testing (AST) including broth microdilution and disc diffusion is widely used to measure AMR in *Shigella* spp. Compared with the phenotypic method, genotypic approaches by detection of AMR genes using PCR method and whole-genome sequencing (WGS) was an alternative solution for AST (11, 12). With the reducing cost of next-generation sequencing (NGS) and increasing accessibility of antibiotic genes, WGS has become a useful tool for genotypic approaches to AMR diagnosis (13). Because NGS-based methods enable the detection of almost all known AMR genes and mutations and the identification of novel variations of AMR determinants, WGS outperforms traditional genotypic approaches for the detection of AMR (14). Additionally, genetic data can be archived indefinitely and reanalysed if new phenotypic AMR determinants are found. The phylogenetic analysis methods based on WGS such as core genome MLST (cgMLST) and core-genome single-nucleotide variant (cgSNV) analysis were used to investigate the genomic epidemiology of *Shigella* and describe the spread of MDR *Shigella* Lineag (15, 16).

Open online databases for antimicrobial resistance genes provide a method to predict the AMR without the need for knowledge of AMR determinants and bioinformatics skills. To date, more than 40 freely available bioinformatics resources have been used to detect AMR determinants in DNA or amino acid sequence data, such as ResFinder and Comprehensive Antimicrobial Resistance Database (17–19). Several studies have established strong antimicrobial genotype-phenotype correlations based on ResFinder and CARD, including S. sonnei, E. coli, Campylobacter, and nontyphoid Salmonella (12, 20, 21). However, our literature review showed that there were only few phenotypic AST studies of S. flexneri isolates based on WGS and comparison studies of the phenotypic and genotypic AST of S. sonnei and S. flexneri isolates from China. The objective of this study was to identify AST characteristics based on genomic sequences of 218 *Shigella* isolates from 2005 to 2016 in Taiyuan city and analyze the correlation between genotypic and phenotypic antibiotic-resistance profiles of *Shigella* spp., especially for fluoroquinolone, macrolides, and third-generation cephalosporins.

## RESULTS

### Genotypic MDR characteristics.

MDR was observed in 96.79% (*n* = 211) *Shigella* isolates in Taiyuan city; 99.08% (*n* = 108) of S. flexneri were MDR isolates, and 94.50% of S. sonnei isolates were MDR. Among these MDR isolates, 95.87%, 48.62%, 27.52%, and 18.81% were resistant to ≥4, ≥5, ≥6, and ≥7 classes of antimicrobials, respectively. The most common MDR patterns in S. flexneri were AMP/NAL/CHL/TET (*n* = 37) and AMP/NAL/CIP/CHL/TET (*n* = 36). AMP/NAL/GEN/TET (*n* = 52) was the dominant MDR pattern in S. sonnei isolates, followed by AMP/FEP/CTX/NAL/GEN/TET (*n* = 11) and AMP/FEP/CTX/NAL/AZM/GEN/TET (*n* = 11) (Table 1).

**TABLE 1:** Drug resistance rate of *Shigella* isolates^*a*^

Antibiotic	S. flexneri (*n* = 109)	S. sonnei (*n* = 109)	Total (*n* = 218)	χ^2^	*P* value
AMP	99.08%	94.50%	96.79%	3.69	0.06
CTX	16.51%	42.20%	29.36%	17.34	<0.01
CAZ	7.34%	11.01%	9.17%	0.88	0.35
FEP	6.42%	42.20%	24.31%	37.92	<0.01
NAL	100.00%	100.00%	100.00%	-	-
CIP	56.88%	16.51%	36.70%	32.23	<0.01
GEN	11.93%	83.49%	47.71%	111.90	<0.01
CHL	94.50%	0.00%	47.25%	195.25	<0.01
TET	85.32%	95.41%	90.37%	6.37	0.01
AZM	10.09%	21.10%	15.60%	5.02	0.03

a AMP, ampicillin; CTX, cefotaxime; CAZ, ceftazidime; FEP, cefepime; NAL, nalidixic acid; CIP, ciprofloxacin (0.0.5 mg/L); GEN, gentamicin; CHL, chloramphenicol; TET, tetracycline; AZM, azithromycin.

### Antimicrobial resistance genes identified in *Shigella* spp.

Thirty-one types of resistance genes or mutations were identified in 109 MDR S. flexneri isolates, and 17 kinds of drug resistance genes or mutations were detected in S. sonnei.

### Resistance to β-lactams.

Resistance to AMP (96.79%), CTX (29.36%), FEP (24.31%), and CAZ (9.17%) were detected among the 218 *Shigella* isolates (Table 1). The resistance rates for CTX and FEP in S. sonnei were considerably higher than those in S. flexneri (*P < *0.01).

The penicillin-resistance genes—*bla*_OXA_ and *bla*_TEM_—and Extended Spectrum β-lactamases (ESBLs) encoded gene—*bla*_CTX-M_—were the major β-lactams resistance genes detected in 218 *Shigella* isolates. All the β-lactams-resistance isolates contained β-lactams-resistance genes except one AMP- and two CTX-resistant S. flexneri isolates. The *bla*_OXA-1_, found in 100 S. flexneri isolates and *bla*_TEM-1_ found in 20 were the most common penicillin-resistance gene variants detected in S. flexneri isolates. More than one penicillin resistance gene was detected in 16 S. flexneri isolates and *bla*_OXA-1_ with one of the *bla*_TEM_ variants was the most common pattern (Table 2). The ESBLs encoded gene *bla*_CTX-M_ with *bla*_CTX-M-3_, *bla*_CTX-M-14_, *bla*_CTX-M-15_, *bla*_CTX-M-55_, and *bla*_CTX-M-123_ variants were detected in 16 cephalosporins-resistant and one cephalosporins-sensitive S. flexneri isolates. In 109 S. sonnei isolates, the *bla*_TEM-1_ (*n* = 67), *bla*_CTX-M-14_ (*n* = 31), and *bla*_CTX-M-55_ (*n* = 11) were the major β-lactams-resistance genes. Thirty-three AMP-resistant S. sonnei isolates were not coded by penicillinase genes, and only the ESBLs-encoded genes were detected that was different from S. flexneri isolates (Table 2). In the 46 CTX- and FEP-resistant S. sonnei isolates, only one ESBLs encoded gene was detected in all but two isolates that contained only the *bla*_OXA-1_ gene. (Table 2).

**TABLE 2:** The AMR genotypes and phenotypes identified in 218 *Shigella* isolates in Taiyuan city^*a*^

	S. flexneri			S. sonnei		
No. of AMR phenotypes	No. of isolates	Phenotypic combinations	Genotypic combinations	No. of isolates	Phenotypic combinations	Genotypic combinations
1	0			2	NAL	*gyrA*(S83L) (2)
2	1	NAL/TET	ND/*gyrA* (S83L), *parC*(S80I)/*tetB*(1)	4	NAL/TET	*gyrA*(S83L)/*tetA* (4)
3	0			2	AMP/NAL/GEN	*bla_TEM-1_/gyrA*(S83L)*/aac(3)-IId* (2)
4	37	AMP/NAL/CHL/TET	*bla_OXA-1_*/*gyrA*(S83L), *parC*(S80I)/*catI*/*tetB*, *tetR* (32)	52	AMP/NAL/GEN/TET	*bla_TEM-1_*/*gyrA*(S83L)/*aac(3)-IId*/*tetA* (50)
			*bla_OXA-1_*, *bla_TEM-1_*/*gyrA*(S83L), *parC*(S80I)/*catI*/*tetA*, *tetB*, *tetR* (1)			*bla_OXA-1_*/*gyrA*(S83L), *parC*(S80I)/ND/*tetB*, *tetR* (1)
			*bla_OXA-1_*, *bla_TEM-116_*/*gyrA*(S83L), *parC*(S80I)/*catI*/*tetB*, *tetR* (1)			*bla_TEM-1_*, *bla_OXA-1_*/*gyrA*(S83L), *parC*(S80I)/*aac(3)-IId* /*tetA* (1)
			ND/*gyrA*(S83L), *parC*(S80I)/ND/*tetB*, *tetR* (1)			
			*bla_TEM-1_*/*gyrA*(S83L)/ND/*tetB*, *tetR* (1)			
			*bla_OXA-1_*/*gyrA*(S83L), *parC*(S80I)/*catI*/*tetA, tetB*, *tetR* (1)			
	10	AMP/NAL/CIP/CHL	*bla_OXA-1_*/*gyrA*(S83L), *gyrA*(D87N), *parC*(S80I)/*catI* (10)			
	3	AMP/NAL/GEN/TET	*bla_TEM-1_*/*gyrA*(S83L)/*aac(3)-IId*/*tetA* (3)			
	1	AMP/NAL/CIP/TET	*bla_TEM-1_*/*gyrA*(S83L), *gyrA*(D87N), *parC*(S80I), QepA2/*tetB*, *tetR* (1)			
5	36	AMP/NAL/CIP/CHL/TET	*bla_OXA-1_*/*gyrA*(S83L), *gyrA*(D87G), *parC*(S80I)/*catI*/*tetB*, *tetR* (3)	2	AMP/NAL/CIP/GEN/TET	*bla_TEM-1_*/*gyrA*(S83L)/*aac(3)-IId*/*tetA* (2)
			*bla_OXA-1_*/*gyrA*(S83L), *parC*(S80I)/*catI*/*tetB*, *tetR* (4)	1	AMP/NAL/AZM/GEN/TET	*bla_TEM-1_*/*gyrA*(S83L)/*mphA*/*aac(3)-IId*/*tetA* (1)
			*bla_OXA-1_*/*gyrA*(S83L), *parC*(S80I), *QnrS1*/*catI*/*tetB*, *tetR* (1)	1	AMP/FEP/CTX/NAL/TET	*bla_CTX-M-14_*/*gyrA*(S83L)/*tetA* (1)
			*bla_OXA-1_*/*gyrA*(S83L), *parC*(S80I), *QnrS1*/*catI*/*tetA*, *tetB*, *tetR* (1)	1	AMP/FEP/CTX/CAZ/NAL	*bla_CTX-M-55_*/*gyrA*(S83L) (1)
			*bla_OXA-1_*, *bla_TEM-1_*/*gyrA*(S83L), *parC*(S80I), *QnrS1*/*catI*/*tetB*, *tetR* (1)			
			*bla_OXA-1_*/*gyrA*(S83L), *parC*(S80I), *QnrA3, aac(6′)-Ib-cr*/*catI*, catB3/*tetA*, *tetB*, *tetR* (1)			
			*bla_OXA-1_, bla_TEM-1_*/*gyrA*(S83L), *parC*(S80I), *QnrS1*/*catI*, *cmlB1*/*tetB*, *tetR* (1)			
			*bla_OXA-1_*/*gyrA*(S83L), *gyrA*(D87N), *parC*(S80I)/*catI*/*tetB*, *tetR* (22)			
			*bla_OXA-1_*, *bla_TEM-1_*/*gyrA*(S83L), *gyrA*(D87N), *parC*(S80I), *QnrS1*/*catI*/*tetA* (1)			
			*bla_CTX-M-14_*/*gyrA*(S83L)/ND/*tetA* (1)			
	1	AMP/NAL/CHL/GEN/TET	*bla_OXA-1_*, *bla_TEM-1_*/*gyrA*(S83L), *parC*(S80I)/*catI*/*aac(3)-IId*/*tetB*, *tetR* (1)			
	1	AMP/CTX/NAL/CHL/TET	*bla_OXA-1_*, *bla_CTX-M-14_*/*bla_CTX-M-14_*/*gyrA*(S83L), *parC*(S80I)/*catI*, *cmlA5*/*tetB*, *tetR* (1)			
	1	AMP/NAL/CIP/AZM/CHL	*bla_OXA-1_*/*gyrA*(S83L), *gyrA*(D87N), *parC*(S80I)/*mphA*/*catI* (1)			
	2	AMP/CTX/NAL/CIP/CHL	*bla_OXA-1_*/ND/*gyrA*(S83L), *gyrA*(D87N), *parC*(S80I)/*catI* (2)			
6	1	AMP/CTX/NAL/CIP/CHL/TET	*bla_OXA-1_*, *bla_TEM-1_*, *bla_CTX-M-3_*/*gyrA*(S83L), *parC*(S80I), *QnrS1*/*catI*/*tetB*, *tetR* (1)	1	AMP/FEP/CTX/NAL/CIP/TET	*bla_OXA-1_*/ND/ND/*gyrA*(S83L), *parC*(S80I)/ND/*tetB*, *tetR* (1)
	1	AMP/NAL/CIP/CHL/GEN/TET	*bla_OXA-1_*, *bla_TEM-1_*/*gyrA*(S83L), *parC*(S80I), *QnrS1*/*catI*/*aac(3)-IId*/*tetB*, *tetR* (1)	11	AMP/FEP/CTX/NAL/GEN/TET	*bla_TEM-1_*, *bla_CTX-M-14_*/*gyrA*(S83L)/*aac(3)-IId*/*tetA* (9)
	1	AMP/CTX/NAL/CHL/GEN/TET	*bla_OXA-1_*, *bla_CTX-M-14_*/*gyrA*(S83L), *parC*(S80I)/*catI*/*aac(3)-IId*/*tetB*, *tetR* (1)			*bla_OXA-1_*/ND/ND/*gyrA*(S83L), *parC*(S80I)/ND/*tetB*, *tetR* (1)
						*bla_TEM-1_*, *bla_CTX-M-55_*/*gyrA*(S83L)/*aac(3)-IId*/*tetA* (1)
				4	AMP/FEP/CTX/CAZ/NAL/TET	*bla_CTX-M-55_*/*gyrA*(S83L)/*tetA* (4)
7	2	AMP/FEP/CTX/CAZ/NAL/CHL/TET	*bla_OXA-1_*, *bla_CTX-M-55_*/*gyrA*(S83L), *parC*(S80I)/*catI*/*tetB*, *tetR* (2)	11	AMP/FEP/CTX/NAL/AZM/GEN/TET	*bla_CTX-M-14_*/*gyrA*(S83L)/*mphA*/*aac(3)-IId*/*tetA* (11)
	2	AMP/CTX/NAL/CIP/AZM/CHL/TET	*bla_OXA-1_*, *bla_TEM-1_*, *bla_CTX-M-14_*/*gyrA*(S83L), *gyrA*(D87N), *parC*(S80I)/*ermB*/*catI*/*tetB*, *tetR* (2)	1	AMP/FEP/CTX/CAZ/NAL/GEN/TET	*bla_TEM-1_*, *bla*CTX-M-64/*gyrA*(S83L)/*aac(3)-IId*/*tetA* (1)
	1	AMP/CTX/NAL/CIP/AZM/CHL/GEN	*bla_OXA-1_*, *bla_TEM-1_*, *bla_TEM-206_*, *bla_TEM-1_*41, *bla_CTX-M-3_*/*gyrA*(S83L), *gyrA*(D87N), *parC*(S80I)/*mphA*/*catI*/*aac(3)-IId* (1)	5	AMP/FEP/CTX/CAZ/NAL/CIP/TET	*bla_CTX-M-55_*/*gyrA*(S83L)/*tetA* (5)
8	1	AMP/FEP/CTX/CAZ/NAL/CIP/CHL/TET	*bla_OXA-1_*, *bla_TEM-1_*, *bla_TEM-141_*, *bla_TEM-206_*, *bla_CTX-M-55_*/*gyrA*(S83L), *gyrA*(D87G), *parC*(S80I)/*catI*/*tetB*, *tetR* (1)	10	AMP/FEP/CTX/NAL/CIP/AZM/GEN/TET	*bla_CTX-M-14_*/*gyrA*(S83L)/*mphA*/*aac(3)-IId*/*tetA* (10)
	1	AMP/CTX/CAZ/NAL/CIP/AZM/CHL/GEN	*bla_OXA-1_*, *bla_CTX-M-14_*/*bla_CTX-M-14_*/*bla_CTX-M-14_*/*gyrA*(S83L), *gyrA*(D87N), *parC*(S80I)/*mphA*, *ermB*/*catI*/*aac(3)-IId* (1)	1	AMP/FEP/CTX/CAZ/NAL/AZM/GEN/TET	*bla_CTX-M-15_*/*gyrA*(S83L)/*mphA*, *ermB*/*aac(3)-IId*/*tetA* (1)
	1	AMP/FEP/CTX/NAL/CIP/AZM/GEN/TET	*bla_CTX-M-14_*/*bla_CTX-M-14_*/*bla_CTX-M-14_*/*gyrA*(S83L)/*mphA*/*aac(3)-IId*/ND (1)			
	1	AMP/CTX/NAL/CIP/AZM/CHL/GEN/TET	*bla_OXA-1_*, *bla_TEM-1_*, *bla_CTX-M-14_*/*gyrA*(S83L), *gyrA*(D87N), *parC*(S80I)/*mphA*/*catI*, *cmlA6*, *cmlA5*/*aac(3)-IId*/*tetB*, *tetR* (1)			
9	1	AMP/CTX/CAZ/NAL/CIP/AZM/CHL/GEN/TET	*bla_OXA-1_*, *bla_TEM-1_*, *bla_CTX-M-55_*/*gyrA*(S83L), *gyrA*(D87N), *parC*(S80I)/*mphA*, *ermB*/*aac(3)-IIa*/*tetB*, *tetR* (1)			
	2	AMP/FEP/CTX/CAZ/NAL/CIP/AZM/CHL/GEN	*bla_OXA-1_*, *bla_TEM-1_*, *bla_CTX-M-15_*/*gyrA*(S83L), *gyrA*(D87N), *parC*(S80I)/*mphA*, *ermB*/*catI*/*aac(3)- IId* (1)			
			*bla_OXA-1_*, *bla_CTX-M-15_*/*gyrA*(S83L), *gyrA*(D87N), *parC*(S80I)/*mphA*, *ermB*/*catI*/*aac(3)- IId* (1)			
	1	AMP/FEP/CTX/CAZ/NAL/CIP/AZM/CHL/TET	*bla_OXA-1_*, *bla_CTX-M-123_*/*gyrA*(S83L), *gyrA*(D87N), *parC*(S80I)/*mphA*/*catI*/*tetB*, *tetR* (1)			

a AMP, ampicillin; CTX, cefotaxime; CAZ, ceftazidime; FEP, cefepime; NAL, nalidixic acid; CIP, ciprofloxacin (0.5 mg/L); GEN, gentamicin; CHL, chloramphenicol; TET, tetracycline; AZM, azithromycin.

### Resistance to fluoroquinolone-resistance genes.

All *Shigella* isolates were resistant to NAL and 36.70% *Shigella* isolates were resistant to CIP (MIC >0.5 mg/L). S. sonnei (16.51%) had a much lower resistance rate to CIP than S. flexneri (56.88%) (*P < *0.01). The inconsistency between the resistance phenotype and genotype was because no associated resistance genes or mutations were found in the 24 CIP-resistant isolates, including six S. flexneri and 18 S. sonnei isolates (Table 3). The mutations in the *QRDR* gene, including *gyrA* and *parC* genes were detected in most *Shigella* isolates. Five nonsynonymous point mutations at codon 83 (Ser to Leu, S83L); codon 87 (Asp to Asn, D87N or Asp to Gly, D87G); codon 211 (His to Tyr, H211Y); and codon 678 (Asp to Glu, D678E) were detected in the *gyrA* gene. Only the point mutation at codon 80 (Ser to Ile, S80I) was detected in the *parC* gene.

**TABLE 3:** Concordance Between Genotypic and Phenotypic Drug-resistant Profiles of *Shigella* isolates^*a*^

	S. flexneri						S. sonnei						
Antibiotic	Phenotype: Resistant		Phenotype: Susceptible		Sensitivity	Specificity	Phenotype: Resistant		Phenotype: Susceptible		Sensitivity	Specificity	Major resistant gene
	Genotype: resistant	Genotype: susceptible	Genotype: resistant	Genotype: susceptible			Genotype: resistant	Genotype: susceptible	Genotype: resistant	Genotype: susceptible			
AMP^*b*^	107	1	0	1	99.07%	100.00%	103	0	0	6	100.00%	100.00%	*bla_TEM-1_*, *bla_OXA-1_*
CTX	16	2	1	90	88.89%	98.90%	44	2	0	63	95.65%	100.00%	*bla*_CTX-M_
CAZ	8	0	9	92	100.00%	91.09%	12	0	32	65	100.00%	67.01%	*bla*_CTX-M_
FEP	7	0	10	92	100.00%	90.20%	44	2	0	63	95.65%	100.00%	*bla*_CTX-M_
NAL	109	0	0	0	100.00%		109	0	0	0	100.00%		*gyrA* (S83L)
CIP	56	6	0	47	90.32%	100.00%	0	18	0	91	0.00%	100.00%	*gyrA* (S83L, D87N/D87G)
GEN	13	0	2	94	100.00%	97.92%	89	2	1	17	97.80%	94.44%	*aac(3)-IId*
CHL	100	3	0	6	97.09%	100.00%	0	0	3	106		97.25%	*catI*
TET^*c*^	92	1	1^*d*^	15	98.92%	93.75%	104	0	0	5	100.00%	100.00%	*tet*A, *tet*B
AZM	11	0	3	95	100.00%	96.94%	23	0	0	86	100.00%	100.00%	*mphA*, *erm*B
Total	519	13	26	532	97.56%	95.34%	528	24	36	502	95.65%	93.31%	

a AMP, ampicillin; CTX, cefotaxime; CAZ, ceftazidime; FEP, cefepime; NAL, nalidixic acid; CIP, ciprofloxacin (0.5 mg/L); GEN, gentamicin; CHL, chloramphenicol; TET, tetracycline; AZM, azithromycin.

b The drug resistance of S. flexneri is mainly related to *bla_OXA-1_* and/or *bla_TEM-1_*, while the drug resistance of S. sonnei is mainly related to *bla_TEM-1_*.

c The drug resistance of S. flexneri is mainly related to *tetB*, while the drug resistance of S. sonnei is mainly related to *tetA*.

d The results of the two drug resistance databases were different. *tet*D and *tetR* were detected in 16 isolates in the CARD database.

**FIG 1:**
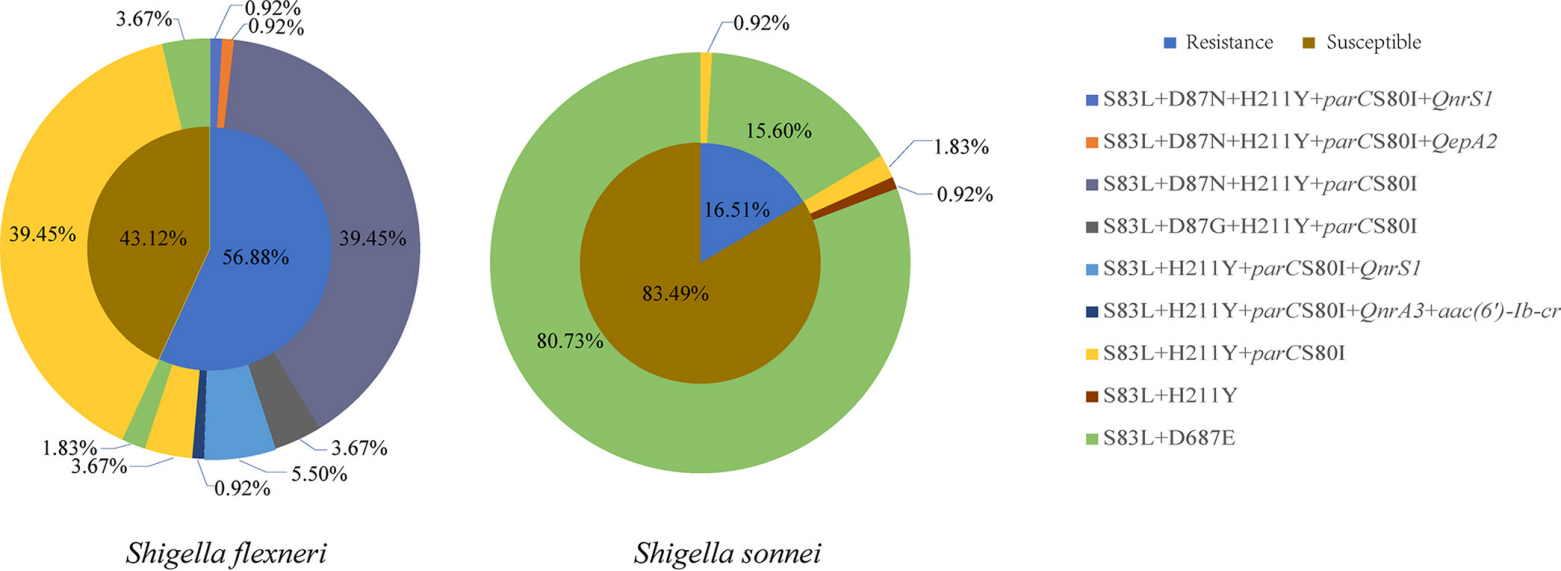
Distribution of CIP-resistant genes and/or mutations in 218 *Shigella* isolates (109 S. flexneri and 109 S. sonnei isolates) from Taiyuan City, Shanxi Province. Inner circle: the resistance rate of *Shigella* isolates. Outer circle: the composition of nine different resistance gene patterns.

Three mutation patterns, including *gyrA* (S83L) with *parC* (S80I), *gyrA* (S83L, D87N) with *parC* (S80I), and *gyrA* (S83L, D87G) with *parC* (S80I) were found in 54, 45, and 4 S. flexneri isolates, respectively. For S. sonnei isolates, the *gyrA* gene with the S83L mutation was detected in all isolates, including three isolates with the *parC* gene mutation S80I at the same time, and the *gyrA* gene with the mutations D87G and D87N were not found (Table 2 and Fig. 1). The plasmid-mediated quinolone resistance (*PMQR*) genes, including *qnrS1*, *qnrA3*, *aac(6′)-Ib-cr*, and *qepA2* were found in nine S. flexneri isolates, and none of the *PMQR* genes were detected in S. sonnei isolates.

There were 45 CIP-resistant (MIC: >0.5 mg/L) S. flexneri isolates containing S83L, D87N, and S80I mutations, including two isolates containing the PMQR genes, *qnrS1* and *qepA2*, respectively. There were 13 CIP-resistant S. flexneri isolates only with the S83L mutation, including seven isolates that had the *PMQR* gene. Neither D87N and D87G mutations nor *PMQR* genes were detected in CIP-sensitive S. flexneri isolates. In 18 CIP-resistant S. sonnei isolates, only the S83L mutation was detected, and one isolate also contained S80I. It was worth mentioning that two CIP-sensitive S. sonnei isolates contained the mutations S83L and S80I (Table 2 and Fig. 1).

The *gyrA* gene mutation H211Y was detected in 103 S. flexneri isolates, and the mutation D678E was found in the other six isolates. In S. sonnei isolates, D678E was the most common mutation that was detected in 105 isolates, and only four isolates contained the H211Y mutation (Fig. 1).

### Resistance to macrolides.

The macrolides-resistant genes detected in *Shigella* isolates were *mphA* and *ermB*, including 10.09% S. flexneri isolates and 21.10% S. sonnei AZM-resistant isolates. The *mphA* genes were detected in 12 S. flexneri isolates and 23 S. sonnei isolates, and *arm* genes were detected in six S. flexneri isolates and one S. sonnei isolate. Five isolates had AZM-resistant phenotype with an MIC >256 mg/L, including four S. flexneri and one S. sonnei isolate which contained both the *mphA* and *ermB* genes. The AZM-resistant phenotype with an MIC value of >16 mg/L was detected in seven S. flexneri and 22 S. sonnei isolates carrying the *ermB* or *mphA* gene. For three S. flexneri isolates only carrying the *mphA* gene, the sensitive phenotype of AZM with an MIC ≤8 mg/L was detected (Table 2).

### Resistance to TET, phenicols, and aminoglycosides.

The resistance rate of 218 *Shigella* isolates with TET was 90.37%, and S. sonnei isolates had higher resistance rate than S. flexneri isolates (*P < *0.05) (Table 1). The *tet* gene was the major tetracycline-resistant gene in both S. flexneri and S. sonnei; however, the variants detected in the two species were different. In S. flexneri isolates, *tetR* (*n* = 102) was detected, followed by *tetB* (*n* = 86), *tetA* (*n* = 13), and *tetD* (*n* = 16). *tetA* (*n* = 101), *tetB* (*n* = 3), and *tetR* (*n* = 3) were detected in S. sonnei isolates. It is worth mentioning that *tetA*, *tetB*, and *tetR* were present simultaneously in six S. flexneri isolates; except one S. flexneri isolate, all the TET-resistant *Shigella* isolates carried the *tet* genes.

No S. sonnei isolate was resistant to CHL, but the resistance rate of S. flexneri isolates to CHL was 94.50% (Table 1). Five types of phenicols-resistant genes were detected in S. flexneri isolates, and *catI* (*n* = 100) was the most abundant, which was also the only phenicol-resistant gene detected in five S. sonnei isolates. The *catI* gene was detected in 100 phenicol-resistant S. flexneri isolates; however, three phenicols-resistant S. flexneri isolates did not carry the *cat*I gene.

The resistance rate for GEN of aminoglycoside antibiotics in S. sonnei isolates was considerably higher than that in S. flexneri isolates (*P < *0.01) (Table 1). The *aac(3)-IIb* was the major GEN-resistant gene that was detected in 89 GEN-resistant and one GEN-sensitive S. sonnei isolate. This gene was also detected in 13 GEN-resistant and two GEN-sensitive S. flexneri isolates. The GEN-resistant gene, *aac(3)-IIa*, was also detected in one GEN-resistant S. flexneri isolate.

### Correlation of phenotypic and genotypic resistance.

A total of 2180 phenotypic tests (10 antimicrobials with 218 *Shigella* isolates) were used to detect the correlation of phenotypic and genotypic resistance of *Shigella* isolates in Taiyuan city. A total of 1051 out of 1090 genotypic tests for S. flexneri isolates were consistent with the phenotypes, 23 types of resistance genes or mutations were identified in 109 MDR S. flexneri isolates. Seventeen drug-resistance genes or mutations were detected in S. sonnei isolates, and 1030 genotypes for S. sonnei isolates out of 1090 genotypic tests were consistent. This resulted in an overall phenotypic and genotype concordance rate of 96.42% and 94.50%, respectively. For S. flexneri isolates, the corresponding resistant genes or mutations were detected in 519 out of 532 resistant phenotypic results with a sensitivity of 97.56%. The genotypic AMR test showed a specificity of 95.34%, for the resistance genes or mutations were detected in 26 of 558 phenotypic sensitivity test results. These resistance genes included 20 β-lactams, three macrolides, two aminoglycosides, and one TET-resistant encoding gene (Table 3). The sensitivity and specificity for the genotypic AMR test were 95.65% and 93.31%, respectively, for S. sonnei isolates. The corresponding resistance genes or mutations were detected in 528 of 552 phenotypic-resistant results of S. sonnei isolates; however, 36 resistance genes were detected among the 538 phenotypic susceptibility results. It is worth noting that the genotypic results were consistent with ResFinder and CARD databases, except for some TET-resistant genes (Table 3).

### Population structure.

**FIG 2:**
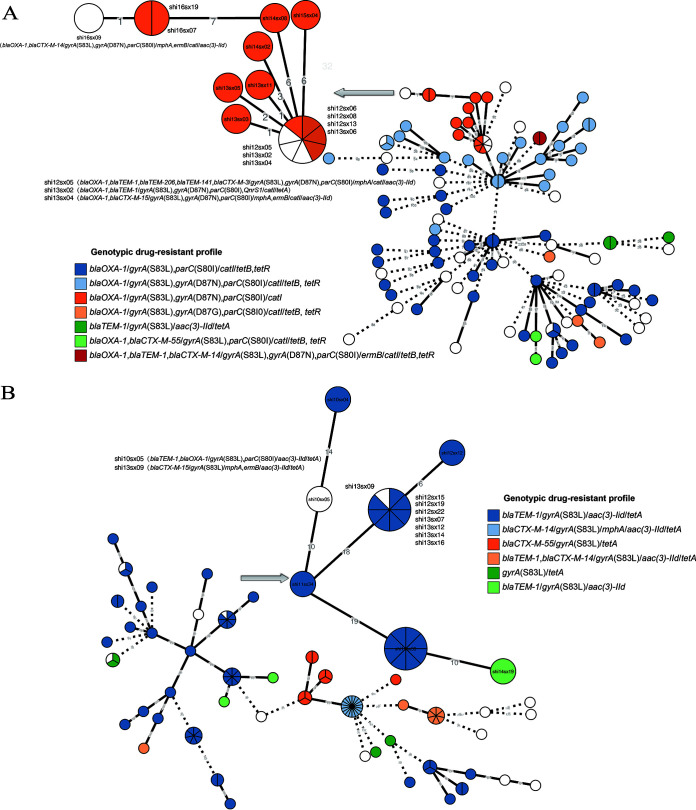
Minimum spanning trees built from the core genome allelic profiles of 109 S. flexneri (A) and 109 S. sonnei isolates (B). The nodes show the number of the strains with pie chart color-coded by genotypic drug-resistant profiles. The allele numbers between the different clusters were labeled on the branches and the branches longer than 20 were hided. The clusters with the isolates belonging to the same cluster with different antibiotic-resistance gene patterns were partial enlarged and the isolate numbers were labeled with the nodes.

The S. flexneri and S. sonnei isolates were phylogenetically analyzed based on the EnteroBase cgMLST scheme, and the isolates with the same genotypic drug-resistant profile were colored (Fig. 2). Most of the S. flexneri and S. sonnei isolates that had the same genotypic drug-resistant profile belonged to the same cluster within a 20-allele difference (Fig. 2). Some isolates belonged to the same cluster with different AMR gene patterns such as seven S. flexneri isolates, including three isolates (shi12sx05, shi13sx02, and shi13sx04) with different genotypic drug-resistant profiles which had the identified cgMLST type. There was also one isolate (shi16sx09) belonging to this cluster that had a different AMR gene pattern from this cluster (Fig. 2A). The difference in the genotypic drug-resistant profiles between the four isolates and the other isolates of this cluster were the AMR genes, including the *bla*_TEM_, *bla_CTX_*, *mphA*, and *ermB* genes located on the plasmid or MDR islands, i.e., the *Shigella*-resistance locus (SRL). The same was observed in some S. sonnei isolates. The isolate (shi13sx09) with the same cgMLST type of seven S. sonnei isolates, had a different genotypic drug-resistant profile without *mphA* and *ermB* genes (Fig. 2B).

## DISCUSSION

Antimicrobial resistance in *Shigella* spp. especially S. flexneri and S. sonnei has become a major public health concern. The AMR patterns of *Shigella* spp. are geographically distinct across the world and in China, and the AMR patterns were also discrepant between S. flexneri and S. sonnei. Obtaining the drug-resistance patterns in distinct regions will help to gain a better understanding of the drug-resistance mechanism of *Shigella* spp. In this study, we found that all the *Shigella* isolates were resistant to NAL. The resistance rate of NAL was much higher in S. flexneri isolates isolated in Jiangsu province (90.50%) from 2006 to 2011 (27), and similar to the S. flexneri and S. sonnei isolates in Shanghai City (96.40%) isolated from 2010 to 2015 (28). For CIP, S. flexneri isolates isolated in Taiyuan city had a much lower resistance rate than S. sonnei, and the resistance rate of S. sonnei was higher than the isolates isolated from Shanghai City (28) and lower than the isolates in England and Wales, 2015 (29). The S. sonnei isolates in Taiyuan city had similar resistance rate as the S. sonnei isolates of Shanghai city to third-generation cephalosporins and CTX. However, the resistance rate of CTX was much higher than that of the S. flexneri isolates isolated from the same city, and the S. sonnei isolates of England and Wales (28, 29).

To effectively treat shigellosis and fully comprehend the genesis and evolution of AMR, it is crucial to be able to reliably identify resistant bacterial isolates and particular AMR genes. In this study, we analyzed the concordance between genotypic and phenotypic drug-resistant profiles of *Shigella* isolates isolated from Taiyuan city based on genomic information. Fluoroquinolones are first-line antimicrobial agents used for the clinical treatment of *Shigella* infection. Mutations in the *QRDR* and *PMQR* genes were detected in fluoroquinolone-resistant *Shigella* isolates, and the *gyrA* gene mutation S83L and *parC* gene mutation S80I were the most popular resistance gene pattern in *Shigella* isolates. The seven S. flexneri isolates carrying *PMQR* genes without D87N or D87G mutations were all CIP-resistant isolates. However, the rate of nonconcordance between genotypic and phenotypic profiles of CIP-resistant S. flexneri isolates by D87N and D87G mutations or *PMQR* gene pattern was 9.68% (6/62). None of the D87N or D87G mutations or *PMQR* genes were detected in S. sonnei isolates. The nonsynonymous point mutations H211Y or D678E of the *gyrA* gene were detected in 218 *Shigella* isolates in Shanxi city. The mutation H211Y was a specific mutation in the pandemic clonal S. flexneri isolates in China from 1997 to 2006 and was likely beneficial to those isolates with quinolone resistance (30). It needs to be further studied whether these mutations conferred quinolone resistance of *Shigella*.

Third-generation cephalosporins and AZM were also used to cure *Shigella* infection. The AMR profiles correlated well between the genotypic and phenotypic results in penicillinase and cephalosporins-resistant S. flexneri isolates. Notably, the ESBLs-encoded gene was detected in 8.91% (*n* = 9) CAZ and 9.80% (*n* = 10) FEP-sensitive S. flexneri isolates, while the *bla*_CTX-M-14_ (*n* = 31) and *bla*_CTX-M-55_ (*n* = 1) genes were present in 32 CAZ-sensitive isolates in S. sonnei. There were 32.04% (*n* = 33) AMP-resistant isolates carrying only the ESBLs encoded genes that were different from S. flexneri isolates. Except for three S. flexneri isolates only carrying the *mphA* gene, all the AZM-resistant *Shigella* isolates carrying the *ermB* or *mphA* gene and the isolates containing both *ermB* and *mphA* genes showed AZM-resistance phenotype with MIC value of >256 mg/L.

There were also some differences in the phenotype-genotype of other classes of antibiotics, which may be caused by the easy loss of plasmids containing drug-resistant genes or acquisition of drug resistance genetic elements (30). The incompleteness of gene fragments caused by the sequencing and missing of novel drug-resistance genes also limited the application of the genotypic AST. For example, β-lactam resistance genes *bla*_TEM_ and *bla*_CTX-M_ are found on the plasmid, as well as the *cat* and *tet* genes are located in the SRL (31), and the loss of those genes might easily result in very major errors (genotypically susceptible but phenotypically resistant). The phylogenetic analysis of S. flexneri and S. sonnei isolates showed that the isolates with the same cluster but different antibiotic-resistance gene patterns were because of the loss or gain of antibiotic-resistance genes located in the plasmids and MDR islands. The other reason that caused the difference in the phenotype-genotype of antibiotics is that some drug-resistance genes could enhance the antibiotic resistance but cannot result in a degree of clinical antibiotic resistance (31). Certainly, the interpretations of AST range may have caused the discrepancy of genotypic and phenotypic AST results of *Shigella* isolates.

This study has some limitations. First, this study was retrospective in design, and the *Shigella* isolates were all isolated from 2005 to 2016 in Taiyuan city. Second, because only 10 kinds of antibiotic drugs were included in the research, this study could not comprehensively describe the genotypic and phenotypic drug-resistant profiles of other *Shigella* isolates.

In summary, we analyzed the concordance between genotypic and phenotypic drug-resistant profiles in *Shigella* isolates isolated in Taiyuan city and found that the two methods correlated well. The drug-resistance patterns and essential drug resistance genes to predict the phenotypic drug-resistant profiles of S. flexneri and S. sonnei isolates in Taiyuan city were distinct. The information on drug resistance and resistance genes in this study can offer more details on the prevalence of drug resistance. Although the drug-resistant genotypes correlated well with phenotypic profiles in the first-generation fluoroquinolones, there were no clear resistance genes and/or mutations to predict the resistance of CIP. The discrepancy of genotypically resistant but phenotypically susceptible isolates also existed in some cephalosporin drugs and AZM-resistant *Shigella* isolates.

## MATERIALS AND METHODS

### Bacterial isolates.

In all, 218 bacterial isolates, including 109 S. flexneri and 109 S. sonnei were isolated from nonrepetitive patients with diarrhea in Taiyuan City, Shanxi Province from 2005 to 2016 (Table S1). All *Shigella* spp. were biochemically identified by the API 20E system (bioMérieux, Marcyl'Étoile, France) and serologically confirmed by slide agglutination test with *Shigella* monovalent antisera (Denka Seiken, Chuo-ku, Japan) according to the manufacturer’s instructions. The verified isolates were stored at −80°C in brain heart infusion broth with 20% glycerol until further analysis.

### Antimicrobial susceptibility testing.

The MICs were determined by broth microdilution method using the Autoscan system (Microscan Walkaway 40 SI, Beckman Coulter, USA) with Neg MIC Panel Type 38 panel following the manufacturer’s instructions. Nine antimicrobial agents were analyzed by broth microdilution method, including chloramphenicol (CHL, 8 to 16 mg/L); tetracycline (TET, 4 to 8 mg/L); ampicillin (AMP, 2 to 16 mg/L); ciprofloxacin (CIP, 0.5 to 2 mg/L); cefotaxime (CTX, 2,8 to 32 mg/L); ceftazidime (CAZ, 1 to 16 mg/L); cefepime (FEP, 2 to 16 mg/L); and gentamicin (GEN, 1 to 8 mg/L). Owing to the absence of nalidixic acid (NAL) and azithromycin (AZM) and the limited range of concentration for CIP of the MIC panel, the disk diffusion method was used to determine the phenotype of *Shigella* isolates with NAL (30 mg) (Oxoid, Hampshire, United Kingdom) and Etest method for AZM (2 to 64 mg/L) and CIP (0.015 to 2 mg/L) (Liofilchem, Roseto Degli Abruzzi, Italy) based on the standard of the Clinical and Laboratory Standards Institute (CLSI) guidelines (22). Escherichia coli ATCC25922 was used as the quality control strain. The phenotypic tests were repeated for those isolates with inconsistent phenotypic and genotypic results. The qualitative interpretations of susceptible (S), intermediate (I), and resistant (R) were determined according to the standard of the CLSI. Multidrug resistance (MDR) was defined as acquired nonsusceptibility to at least one agent in ≥3 antimicrobial classes.

### Genome sequencing.

Wizard Genomic DNA purification kit (Promega, Madison, USA) was used to extract the genomic DNA of *Shigella* spp. according to the manufacturer’s instructions. The Illumina HiSeq 2500 platform (Illumina, San Diego, USA) was used to sequence the DNA library which was produced by the Nextera XT v2 kit (Illumina, San Diego, USA). The adapter and the readers with index and low Q20 scores from the paired-end sequencing data were filtered out using fast 0.20.1. The filtered reads were assembled using SOAPdenovo2 (https://www.sourceforge.net/projects/soapdenovo2/files/SOAPdenovo2/) to produce the draft genomes. The sequencing depth of all *Shigella* isolates were greater than 150×.

### Resistance genes identification.

Two free online databases ResFinder (https://cge.food.dtu.dk/services/ResFinder/) and CARD (https://card.mcmaster.ca/home) were used to identify the AMR genes in the WGS data. For Resfinder, the default parameters included 90% as the threshold for ID and 60% as the minimum length were used. In CARD, the data were analyzed based on select perfect and strict hits only, and nudge ≥95% identity loose hits to strict with RGI (Resistance Gene Identifier) to ascertain whether the reference sequence is present or whether there are nucleotide variations within it. The gene sequences of quinolone resistance determining regions (QRDR) including *gyrA* and *parC* genes were analyzed to detect the relationship between those genes and quinolone-resistant *Shigella* isolates. The full-length sequences of the *gyrA* gene were aligned and analyzed using MEGA 7.0 (http://www.megasoftware.net/) to determine all the nucleotide variations within those sequences.

### Phylogenetic analysis.

cgMLST was performed for *Shigella* isolates using the E. coli/*Shigella*-specific EnteroBase scheme comprising 2513 single-copy orthologous genes (https://enterobase.warwick.ac.uk). The cgMLST alleles obtained by comparing with 2513 genes were used to calculate genetic distances between genomes. The minimum spanning trees of S. flexneri and S. sonnei isolates were constructed based on the number of shared cgMLST alleles and were visualized using GrapeTree (23–26).

### Statistical analysis.

For data analysis, the chi-square test in SPSS statistics software version 26 was performed to determine the significant difference between variables. *P < *0.05 was considered to indicate statistical significance.

### Data availability.

Genomic sequence data used in this study were deposited in NCBI under BioProject number PRJNA903515.

## References

[1] Kotloff KL, Riddle MS, Platts-Mills JA, Pavlinac P, Zaidi AKM. 2018. Shigellosis. Lancet 391:801–812. doi:10.1016/S0140-6736(17)33296-8. 29254859

[2] Baker S, The HC. 2018. Recent insights into Shigella. Curr Opin Infect Dis 31:449–454. doi:10.1097/QCO.0000000000000475. 30048255 PMC6143181

[3] Li S, Sun Q, Wei X, Klena J, Wang J, Liu Y, Tian K, Luo X, Ye C, Xu J, Wang D, Tang G. 2015. Genetic characterization of Shigella flexneri isolates in Guizhou Province, China. PLoS One 10:e0116708. doi:10.1371/journal.pone.0116708. 25617838 PMC4305296

[4] Qu M, Zhang X, Liu G, Huang Y, Jia L, Liang W, Li X, Wu X, Li J, Yan H, Kan B, Wang Q. 2014. An eight-year study of Shigella species in Beijing, China: serodiversity, virulence genes, and antimicrobial resistance. J Infect Dev Ctries 8:904–908. doi:10.3855/jidc.3692. 25022302

[5] Zhao L, Xiong Y, Meng D, Guo J, Li Y, Liang L, Han R, Wang Y, Guo X, Wang R, Zhang L, Gao L, Wang J. 2017. An 11-year study of shigellosis and Shigella species in Taiyuan, China: active surveillance, epidemic characteristics, and molecular serotyping. J Infect Public Health 10:794–798. doi:10.1016/j.jiph.2017.01.009. 28188118

[6] Wang Y, Ma Q, Hao R, Zhang Q, Yao S, Han J, Ren B, Fan T, Chen L, Xu X, Qiu S, Yang H. 2019. Antimicrobial resistance and genetic characterization of Shigella spp. in Shanxi Province, China, during 2006–2016. BMC Microbiol 19:116. doi:10.1186/s12866-019-1495-6. 31142259 PMC6542020

[7] Christopher PR, David KV, John SM, Sankarapandian V. 2010. Antibiotic therapy for Shigella dysentery. Cochrane Database Syst Rev 2010:Cd006784. doi:10.1002/14651858.CD006784.pub4. 20687081 PMC6532574

[8] WHO. 2017. The selection and use of essential medicines. http://www.who.int/medicines/publications/essentialmedicines/en/.

[9] Chang Z, Zhang J, Ran L, Sun J, Liu F, Luo L, Zeng L, Wang L, Li Z, Yu H, Liao Q. 2016. The changing epidemiology of bacillary dysentery and characteristics of antimicrobial resistance of Shigella isolated in China from 2004–2014. BMC Infect Dis 16:685. doi:10.1186/s12879-016-1977-1. 27863468 PMC5116132

[10] Chung The H, Rabaa MA, Pham Thanh D, De Lappe N, Cormican M, Valcanis M, Howden BP, Wangchuk S, Bodhidatta L, Mason CJ, Nguyen Thi Nguyen T, Vu Thuy D, Thompson CN, Phu Huong Lan N, Voong Vinh P, Ha Thanh T, Turner P, Sar P, Thwaites G, Thomson NR, Holt KE, Baker S. 2016. South Asia as a reservoir for the global spread of ciprofloxacin-resistant Shigella sonnei: a cross-sectional study. PLoS Med 13:e1002055. doi:10.1371/journal.pmed.1002055. 27483136 PMC4970813

[11] Katiyar A, Sharma P, Dahiya S, Singh H, Kapil A, Kaur P. 2020. Genomic profiling of antimicrobial resistance genes in clinical isolates of Salmonella Typhi from patients infected with Typhoid fever in India. Sci Rep 10:8299. doi:10.1038/s41598-020-64934-0. 32427945 PMC7237477

[12] Tyson GH, McDermott PF, Li C, Chen Y, Tadesse DA, Mukherjee S, Bodeis-Jones S, Kabera C, Gaines SA, Loneragan GH, Edrington TS, Torrence M, Harhay DM, Zhao S. 2015. WGS accurately predicts antimicrobial resistance in Escherichia coli. J Antimicrob Chemother 70:2763–2769. doi:10.1093/jac/dkv186. 26142410 PMC11606221

[13] Hendriksen R, Bortolaia V, Tate H, Tyson G, Aarestrup F, McDermott P. 2019. Using genomics to track global antimicrobial resistance. JFiph 7:242. 10.3389/fpubh.2019.00242 PMC6737581 31552211

[14] Pfaller MA. 2001. Molecular approaches to diagnosing and managing infectious diseases: practicality and costs. Emerg Infect Dis 7:312–318. doi:10.3201/eid0702.010234. 11294731 PMC2631730

[15] Holt KE, Baker S, Weill FX, Holmes EC, Kitchen A, Yu J, Sangal V, Brown DJ, Coia JE, Kim DW, Choi SY, Kim SH, da Silveira WD, Pickard DJ, Farrar JJ, Parkhill J, Dougan G, Thomson NR. 2012. Shigella sonnei genome sequencing and phylogenetic analysis indicate recent global dissemination from Europe. Nat Genet 44:1056–1059. doi:10.1038/ng.2369. 22863732 PMC3442231

[16] Yassine I, Lefèvre S, Hansen EE, Ruckly C, Carle I, Lejay-Collin M, Fabre L, Rafei R, Clermont D, de la Gandara MP, Dabboussi F, Thomson NR, Weill FX. 2022. Population structure analysis and laboratory monitoring of Shigella by core-genome multilocus sequence typing. Nat Commun 13:551. doi:10.1038/s41467-022-28121-1. 35087053 PMC8795385

[17] Bortolaia V, Kaas RS, Ruppe E, Roberts MC, Schwarz S, Cattoir V, Philippon A, Allesoe RL, Rebelo AR, Florensa AF, Fagelhauer L, Chakraborty T, Neumann B, Werner G, Bender JK, Stingl K, Nguyen M, Coppens J, Xavier BB, Malhotra-Kumar S, Westh H, Pinholt M, Anjum MF, Duggett NA, Kempf I, Nykäsenoja S, Olkkola S, Wieczorek K, Amaro A, Clemente L, Mossong J, Losch S, Ragimbeau C, Lund O, Aarestrup FM. 2020. ResFinder 4.0 for predictions of phenotypes from genotypes. J Antimicrob Chemother 75:3491–3500. doi:10.1093/jac/dkaa345. 32780112 PMC7662176

[18] Zankari E, Hasman H, Cosentino S, Vestergaard M, Rasmussen S, Lund O, Aarestrup F, Larsen M. 2012. Identification of acquired antimicrobial resistance genes. J Antimicrob Chemother 67:2640–2644. doi:10.1093/jac/dks261. 22782487 PMC3468078

[19] Stubberfield E, AbuOun M, Sayers E, O'Connor H, Card R, Anjum M. 2019. Use of whole genome sequencing of commensal in pigs for antimicrobial resistance surveillance, United Kingdom, 2018. JEsbEslmtEcdb 24. doi:10.2807/1560-7917.ES.2019.24.50.1900136. PMC6918588 31847943

[20] McDermott P, Tyson G, Kabera C, Chen Y, Li C, Folster J, Ayers S, Lam C, Tate H, Zhao S. 2016. Whole-genome sequencing for detecting antimicrobial resistance in nontyphoidal Salmonella. Antimicrob Agents Chemother 60:5515–5520. doi:10.1128/AAC.01030-16. 27381390 PMC4997858

[21] Zhao S, Tyson G, Chen Y, Li C, Mukherjee S, Young S, Lam C, Folster J, Whichard J, McDermott PJA. 2016. Whole-genome sequencing analysis accurately predicts antimicrobial resistance phenotypes in Campylobacter spp. Appl Environ Microbiol 82:459–466. doi:10.1128/AEM.02873-15. 26519386 PMC4711122

[22] Institute. CaLS. Performance standards for antimicrobial susceptibility testing. M100 standard, 29th ed Clinical and Laboratory Standards Institute, Wayne, PA. 10.1128/JCM.00213-21 PMC8601225 34550809

[23] Zhou Z, Alikhan NF, Mohamed K, Fan Y, Achtman M, Agama Study Group. 2020. The EnteroBase user's guide, with case studies on Salmonella transmissions, Yersinia pestis phylogeny, and Escherichia core genomic diversity. Genome Res 30:138–152. doi:10.1101/gr.251678.119. 31809257 PMC6961584

[24] Alikhan NF, Zhou Z, Sergeant MJ, Achtman M. 2018. A genomic overview of the population structure of Salmonella. PLoS Genet 14:e1007261. doi:10.1371/journal.pgen.1007261. 29621240 PMC5886390

[25] Zhou Z, Alikhan NF, Sergeant MJ, Luhmann N, Vaz C, Francisco AP, Carriço JA, Achtman M. 2018. GrapeTree: visualization of core genomic relationships among 100,000 bacterial pathogens. Genome Res 28:1395–1404. doi:10.1101/gr.232397.117. 30049790 PMC6120633

[26] Zhou Z, Lundstrøm I, Tran-Dien A, Duchêne S, Alikhan NF, Sergeant MJ, Langridge G, Fotakis AK, Nair S, Stenøien HK, Hamre SS, Casjens S, Christophersen A, Quince C, Thomson NR, Weill FX, Ho SYW, Gilbert MTP, Achtman M. 2018. Pan-genome analysis of ancient and modern Salmonella enterica demonstrates genomic stability of the invasive para C lineage for millennia. Curr Biol 28:2420–2428.e10. doi:10.1016/j.cub.2018.05.058. 30033331 PMC6089836

[27] Qin T, Bi R, Fan W, Kang H, Ma P, Gu B. 2016. Novel mutations in quinolone resistance-determining regions of gyrA, gyrB, parC and parE in Shigella flexneri clinical isolates from eastern Chinese populations between 2001 and 2011. Eur J Clin Microbiol Infect Dis 35:2037–2045. doi:10.1007/s10096-016-2761-2. 27620866

[28] Zhang WX, Chen HY, Tu LH, Xi MF, Chen M, Zhang J. 2019. Fluoroquinolone resistance mechanisms in Shigella Isolates in Shanghai, China, between 2010 and 2015. Microb Drug Resist 25:212–218. doi:10.1089/mdr.2018.0113. 30307807

[29] Sadouki Z, Day MR, Doumith M, Chattaway MA, Dallman TJ, Hopkins KL, Elson R, Woodford N, Godbole G, Jenkins C. 2017. Comparison of phenotypic and WGS-derived antimicrobial resistance profiles of Shigella sonnei isolated from cases of diarrhoeal disease in England and Wales, 2015. J Antimicrob Chemother 72:2496–2502. doi:10.1093/jac/dkx170. 28591819

[30] Zhang N, Lan R, Sun Q, Wang J, Wang Y, Zhang J, Yu D, Hu W, Hu S, Dai H, Du P, Wang H, Xu J. 2014. Genomic portrait of the evolution and epidemic spread of a recently emerged multidrug-resistant Shigella flexneri clone in China. J Clin Microbiol 52:1119–1126. doi:10.1128/JCM.02669-13. 24452172 PMC3993495

[31] Ye C, Lan R, Xia S, Zhang J, Sun Q, Zhang S, Jing H, Wang L, Li Z, Zhou Z, Zhao A, Cui Z, Cao J, Jin D, Huang L, Wang Y, Luo X, Bai X, Wang Y, Wang P, Xu Q, Xu J. 2010. Emergence of a new multidrug-resistant serotype X variant in an epidemic clone of Shigella flexneri. J Clin Microbiol 48:419–426. doi:10.1128/JCM.00614-09. 19955273 PMC2815595

