# Supplementary material for: Concordance between Genotypic and Phenotypic Drug-Resistant Profiles of Shigella Isolates from Taiyuan City, Shanxi Province, China, 2005 to 2016
Source: Microbiol Spectr. 2023 May 30;11(3):e00119-23. doi: 10.1128/spectrum.00119-23 (PMC10269460; doi:10.1128/spectrum.00119-23)
Supplement: Supplemental file 1 — Table S1. Download spectrum.00119-23-s0001.pdf, PDF file, 0.06 MB [file spectrum.00119-23-s0001.pdf]

Table S1 Strains information used in this study

Year	No. of *S. flexneri*	*No. of S. sonnei*	Total
2005	7	0	7
2006	28	5	33
2007	6	15	21
2008	10	1	11
2009	5	3	8
2010	3	7	10
2011	7	21	28
2012	11	10	21
2013	10	12	22
2014	14	8	22
2015	4	3	7
2016	4	24	28
Total	109	109	218
